# Supplementary figures and images for: Decreased R:FR Ratio in Incident White Light Affects the Composition of Barley Leaf Lipidome and Freezing Tolerance in a Temperature-Dependent Manner
Source: Int J Mol Sci. 2020 Oct 13;21(20):7557. doi: 10.3390/ijms21207557 (PMC7593930; doi:10.3390/ijms21207557)

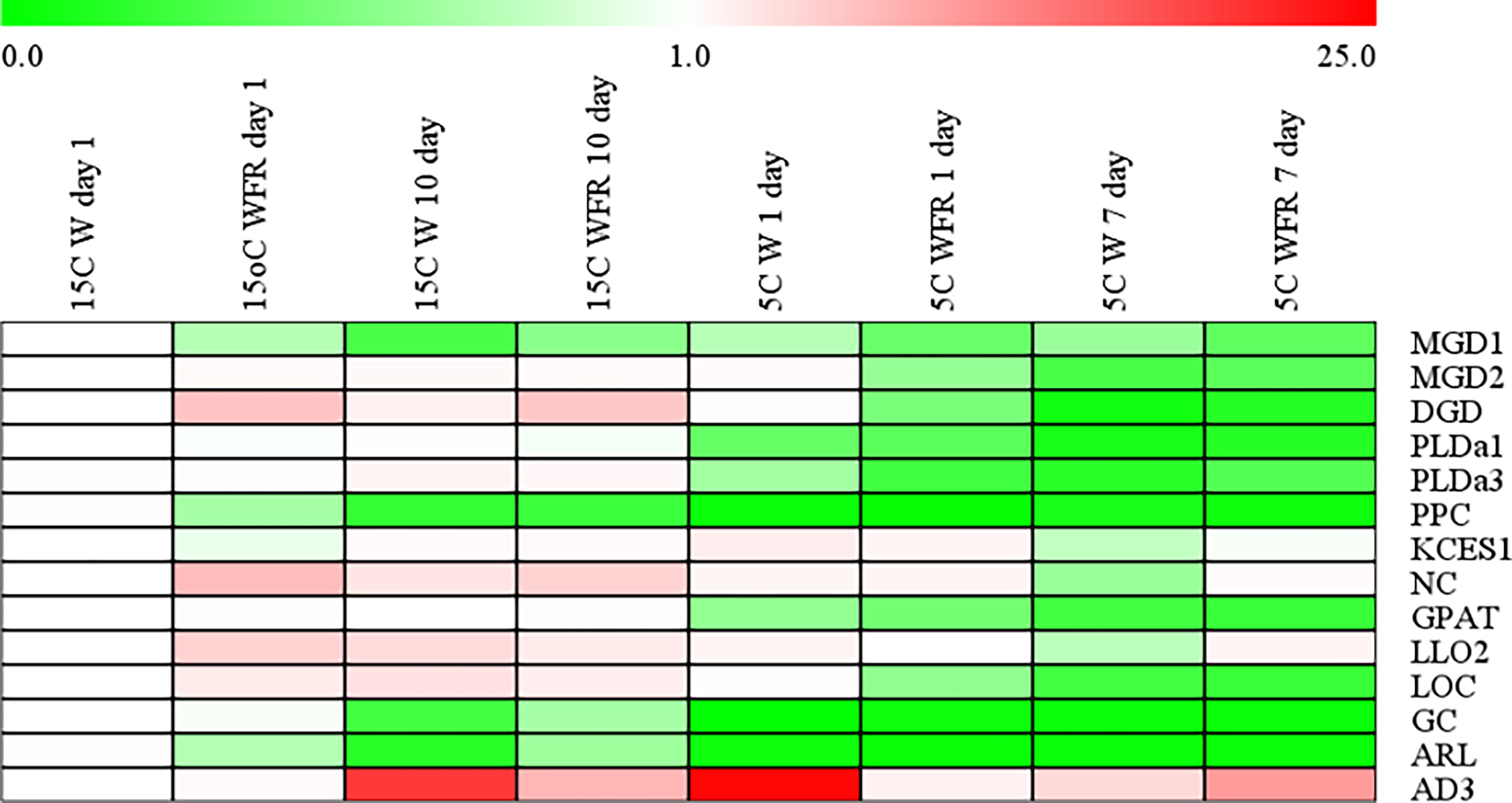

Supplement: Supplementary file 1 [file ijms-21-07557-s001.zip › Supplementary_Fig2.jpg]

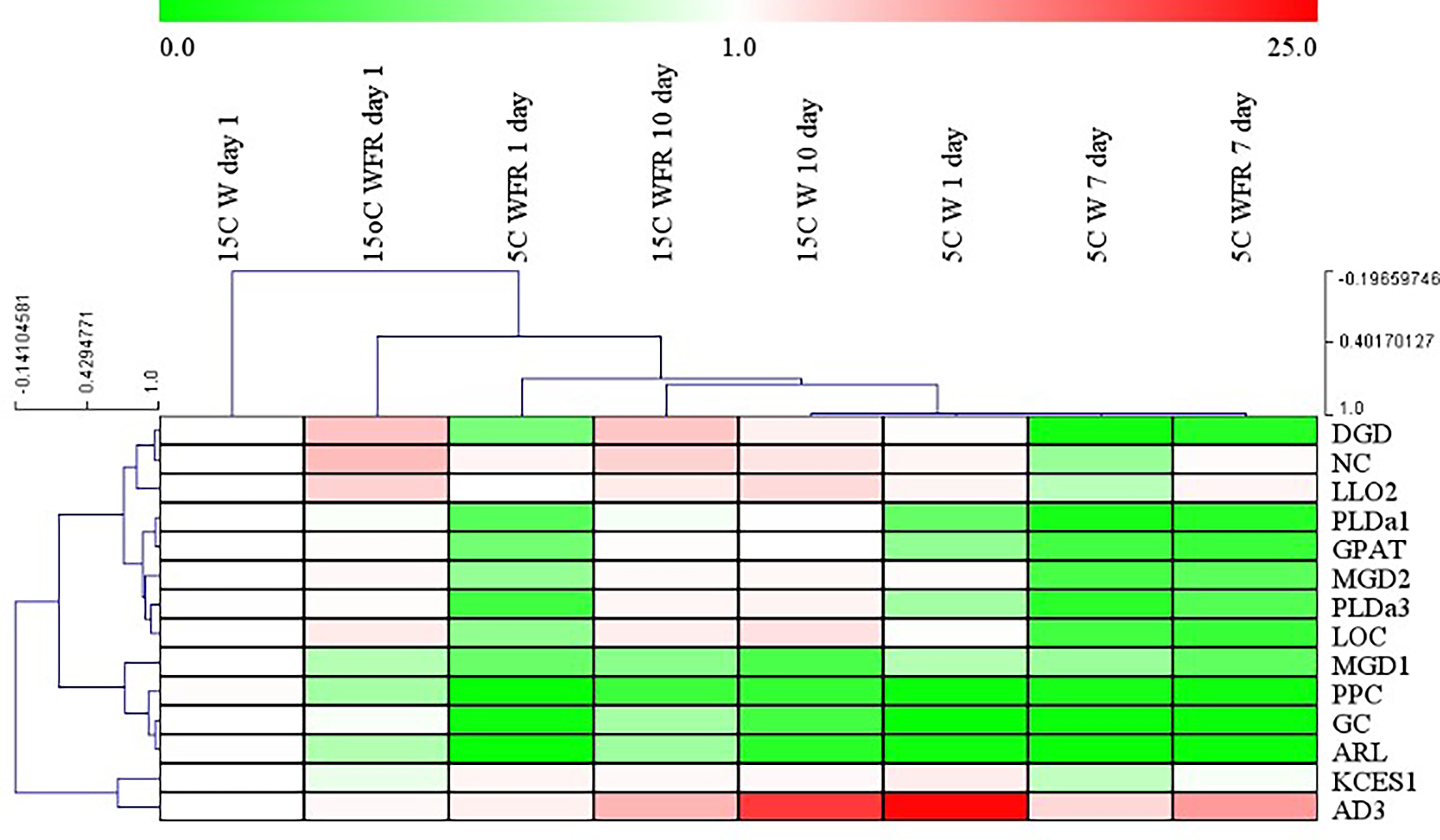

Supplement: Supplementary file 1 [file ijms-21-07557-s001.zip › Supplementary_Fig_3.jpg]

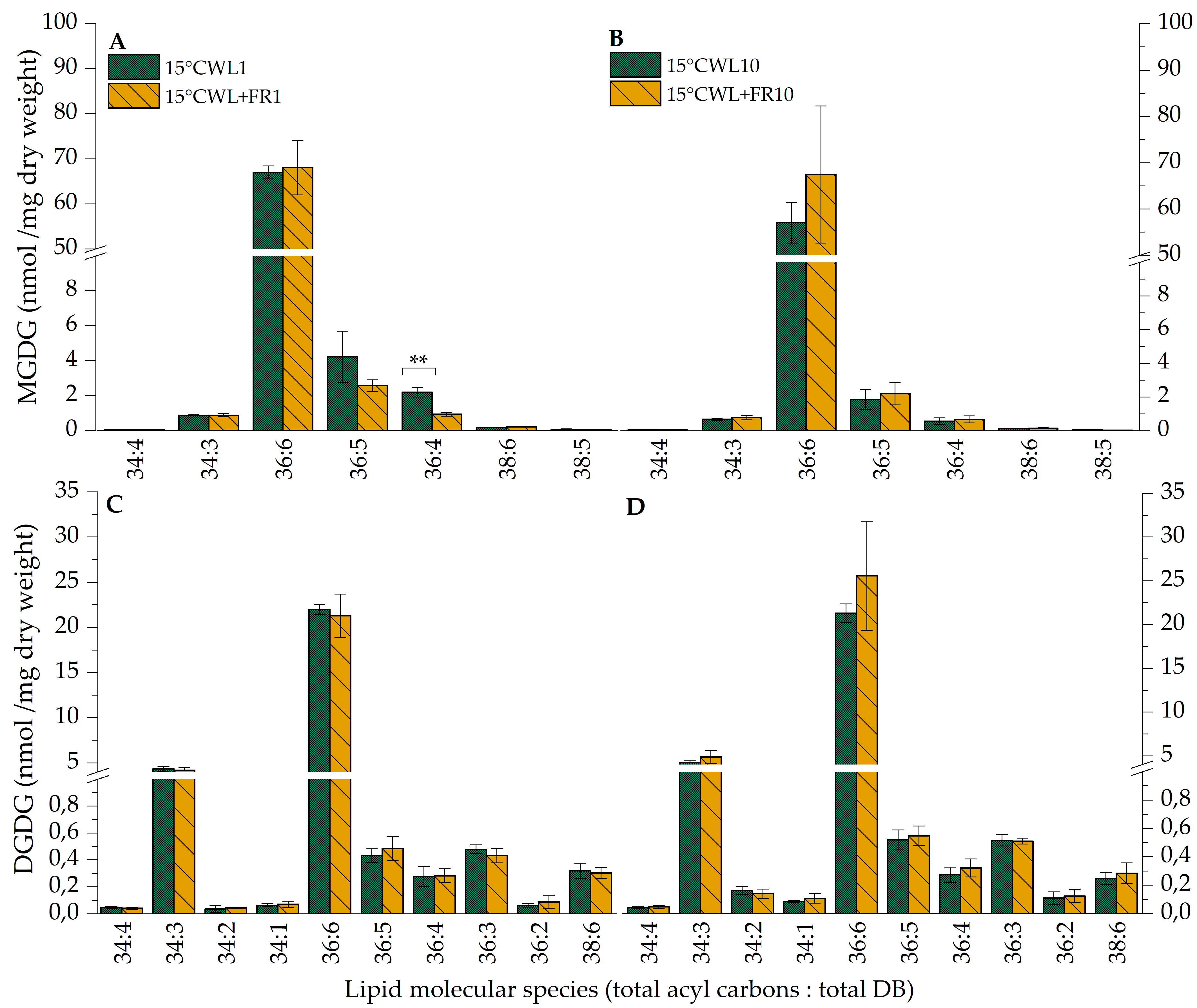

Supplement: Supplementary file 1 [file ijms-21-07557-s001.zip › Supplementary_Fig_4.tif]

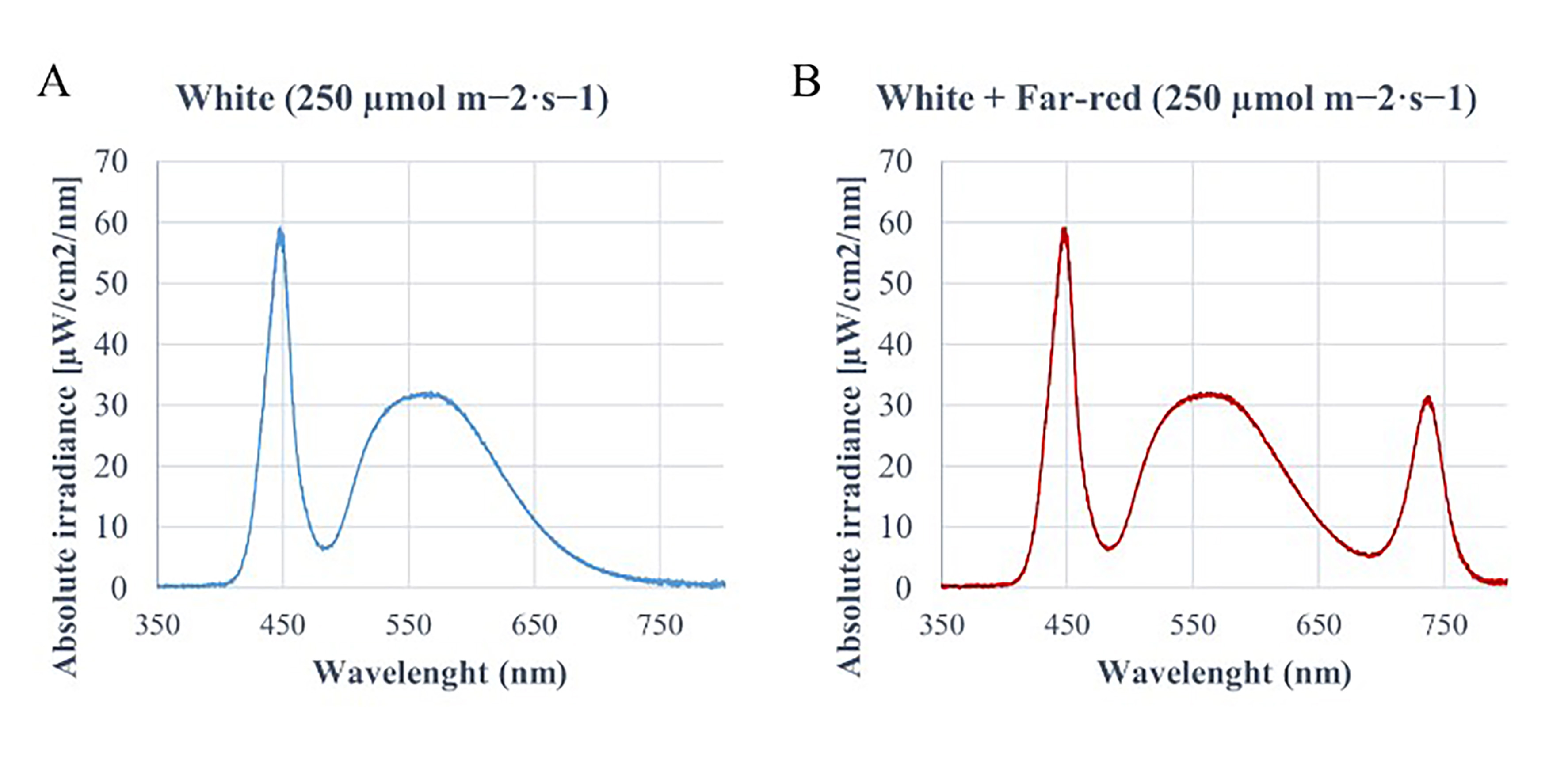

Supplement: Supplementary file 1 [file ijms-21-07557-s001.zip › Supplementary_Fig1.jpg]
